# Use of PerClot^®^ in head and neck surgery: a Scottish centre experience

**DOI:** 10.1007/s00405-020-06247-6

**Published:** 2020-08-06

**Authors:** Kanishka Rao, Anas Gomati, Edwin Yuen Hao Tong, Kim W Ah-See, Muhammad Shakeel

**Affiliations:** Department of Otolaryngology-Head and Neck Surgery, Aberdeen Royal Infirmary, Aberdeen, AB25 2ZN Scotland, UK

**Keywords:** PerClot^®^, Head and neck Surgery, Haemostatic agent

## Abstract

**Objective:**

PerClot^®^ is a biocompatible, polysaccharide haemostatic system recommended for surgical procedures. It is an absorbable modified polymer that is non-pyrogenic and is derived from purified plant starch. Our goal was to evaluate the safety, efficacy and usefulness of PerClot^®^ in head and neck surgery (H&N) in our department.

**Methods:**

All patients who received PerClot^®^ after their neck operation over 1-year period (2019–2020) were prospectively investigated. The information collected included demographics, admission and discharge dates, type of operation, operative details, postoperative complications and their management. The data were collected and analysed using Excel.

**Results:**

A total of 57 patients (males = 26, females = 31) with mean age of 51 (range 19–83) were identified. None of the patients developed primary or secondary haemorrhage. Ten patients suffered from post-operative wound complications (18%). Wound infection was noticed in 9/57 (16%) of patients. 1/57 patients had seroma.

**Conclusions:**

PerClot^®^ is safe, effective in reducing the postoperative bleeding and would appear to be useful in head and neck surgery with minimal adverse effects.

## Introduction

Postoperative haemorrhage is one of the principal concerns in head and neck surgery. It can present significant perioperative morbidity [1], therefore leading to further surgical intervention, increased risk of infection and prolonged hospital stay [2]. The conventional techniques for haemostasis are pressure, suture ligation and electrocoagulation. In the recent years, a multitude of topical haemostatic agents have been adopted and widely used across the variety of surgical specialities. Although some complications have been reported, they have been generally considered safe [3].

Currently, in Otolaryngology and Head and Neck surgery (H&Ns), the regular use of haemostatic sealants is not well established as compared to other surgical specialities such as neurosurgery, where in certain centres, up to 80% of all cranial and spinal surgery are using these agents [4].

To our knowledge, only one published study in the English literature utilises the usage of PerClot^®^ as a haemostatic agent in head and neck surgery [5], with a lower-case cohort than our current work.

PerClot^®^ Polysaccharide Hemostatic System (PerClot^®^ PHS) is a unique, absorbable powder haemostat. It is a plant-based haemostat manufactured by Starch Medical Inc. (San Jose, California) [6]. It consists of dry, sterile, polysaccharide particles manufactured from purified plant starch using proprietary modification processes. It has been proposed that PerClot^®^ can improve wound healing by means of increasing in the activity of fibroblasts, leading to increased release of TGF-β1 [7].

The aim of this study was to see the effectiveness of PerClot^®^ as a haemostatic agent in head and neck surgery and to analyse any observed adverse features.

## Methods

This retrospective analysis of a prospectively maintained departmental database was conducted at a Scottish tertiary centre. PerClot^®^ was introduced to our department in February 2019. Therefore, we are analysing the data entered covering a period of 1 year, February 2019–February 2020.

In all patients, who underwent a head and neck procedure, PerClot^®^ was used. Clear data entry of this was documented in the operation note and the data were also entered in our departmental excel data sheet. Further details were obtained from the electronic records including further follow-up clinics.

Data collection included patient demographics, type of procedure, usage of surgical drains, post-operative complications; including haematoma, seroma, wound infection and duration of hospital stay.

The audit was registered with the institutional clinical effectiveness department.

Microsoft excel was used to collect, analyse and tabulate these data.

## Results

Between February 2019 and 2020, a total of 57 patients received PerClot^®^ as a haemostatic agent in the surgical site before closure. There were 31 females and 26 males with an average age of 51 years (range 19–83). The patients underwent a wide range of head and neck operations as detailed in (Table 1).

**Table 1:** Details of operations patients underwent

Operation	Number
Per Clot group = 57 patients	
Hemithyroidectomy	13
Partial parotidectomy	14
Neck node excision and incision biopsy	11
Sistrunk procedure	4
Neck dissection	4
Total thyroidectomy	3
Neck lipoma excision	3
Branchial cyst excision	2
Submandibular gland excision	1
Transoral excision of left parapharyngeal mass	1
Total thyroidectomy + neck dissection + sternotomy	1

The majority of these procedures were carried out under general anaesthesia with local anaesthetic infiltration (*n* = 50 patients), while seven patients underwent their surgery using local anaesthetic alone. Of the 57 patients, 17 were discharged home the same day, 24 stayed one night, eight patients two nights, four patients three nights, and four patients stayed between 4 and 12 nights in hospital after the surgery.

Of the 57, only 16 patients required insertion of a suction drain after partial parotidectomy five, neck dissection four, Sistrunk procedure four and thyroidectomy three.

None of the patients in the study group developed primary or secondary haemorrhage. No allergic reaction to Per Clot was observed in our patient cohort. However, ten out of the fifty-seven (10/57) patients suffered post-operative complication (18%). Postoperative wound infection was noticed in 9 (16%) of our patients, while one patient developed seroma.

Out of the 16 patients who required a neck drain insertion, two patients developed post-operative wound infection treated with oral antibiotics in the community. One patient developed seroma which was managed by needle aspiration upon clinical review.

In the remaining 41 patients, no drain was used. In addition to PerClot^®^, seven patients also received surgicel. Out of this series, two patients developed post-operative wound infections. The majority of our patients’ symptoms resolved with oral antibiotics. However, one patient (1/7) required further admission for intra-venous antibiotic treatment.

## Discussion

Local haemostatic agents have been available for several years. The utilisation of haemostatic agents has increased expeditiously over the last decade [2]. A few studies in the literature have reported various results on the usage of intraoperative haemostatic agents in head and neck surgery. Browder IW and Litwin MS et al., in 1986, used an absorbable collagen for haemostasis in thyroid surgery [8].

In a study conducted by A Ujam et al., prospectively trialed Floseal^®^, as a haemostatic agent in 42 various head and neck surgical procedures, there were no adverse features reported other than, two patients developing post-operative surgical haematoma. Floseal^®^ was concluded as a safe adjunct to be used in head and neck surgery [9].

In a retrospective study by M.Bannister et al., 17 patients underwent submandibular gland excision and during intra-operative haemostasis, Surgiflo^®^ was used. Neither side effects nor complications were noted, and good haemostasis was reported [3].

PerClot^®^ is designed to control bleeding over large surfaces and localised bleeding areas. It instantly attracts the fluid from blood forming a gelled matrix, reducing further bleeding. It accelerates the intrinsic clotting cascade with no inherent risk of adverse events after the clot is formed. PerClot^®^ will be completely resorbed in a few days via natural enzyme activity. Unlike other hemostats that continue to swell after application, PerClot^®^ particles reach their maximum volume immediately upon contact with blood or other fluids.

Different studies have evaluated the effectiveness of PerClot^®^ as a topical haemostatic agent. In a study by Yanxia Wang et al., on the effect of PerClot^®^ in the healing of full thickness skin wounds in rats, PerClot^®^ was found to significantly accelerate wound healing by increasing the activity of fibroblasts and increased the release of TGF-β1 [7].

V.Tscholl et al. evaluated the effect of PerClot^®^ application in patients with high bleeding risk factors undergoing cardiac rhythm device implantation. The results from this study reported that PerClot^®^ did not seem to decrease the frequency of haematoma [2].

In a study conducted by Thomas von Ahnen et al., they compared the intraoperative application of PerClot^®^ with conventional haemostatic procedure after thyroid resection. The outcome parameters were postoperative bleeding, the drainage volume 24 h postoperatively and adverse events. This study concluded that PerClot^®^ had no advantage over conventional hemostasis technique in thyroid surgery, while reported to be safe and well tolerated [5].

In a prospective study conducted by Helmut Mair et al., PerClot^®^ was used on 21 patients undergoing coronary surgery requiring median sternotomy to help control sternal bleeding. Based on the observation in this case series, it was concluded that PerClot^®^ exhibits a greater haemostatic efficacy. It also reported a lesser time to haemostasis and increased the clot strength [6].

There have been various studies that have published regarding the safety and effectiveness of haemostatic agents in operations in head and neck and we know only one that relates to the use of PerClot^®^, the study conducted by Thomas von Ahnen et al. [5]

The ideal haemostatic agent should be inexpensive, bioabsorbable, effective, safe and easy to use. On reviewing the literature, there is some evidence of adverse events reported for haemostatic agents such as wound infection, allergic reactions, nerve entrapment [10] and can cause misleading postoperative imaging interpretation [11].

The haemostatic effect of PerClot^®^ has been proven and until now, no adverse events occur on using PerClot^®^ [12].

The results of the present study are contradictory to these findings and confirm the occurrence of increase in risk of post-operative wound infections with the use of polysaccharide particles. There are a few advantages in comparison to other haemostatic agents in whic, PerClot^®^ has a simple way of application and is readily available to apply to operative site with no need of preparation before instillation. Applying PerClot^®^ to the whole surgical wound cavity could easily be achieved.

**Fig. 1:**
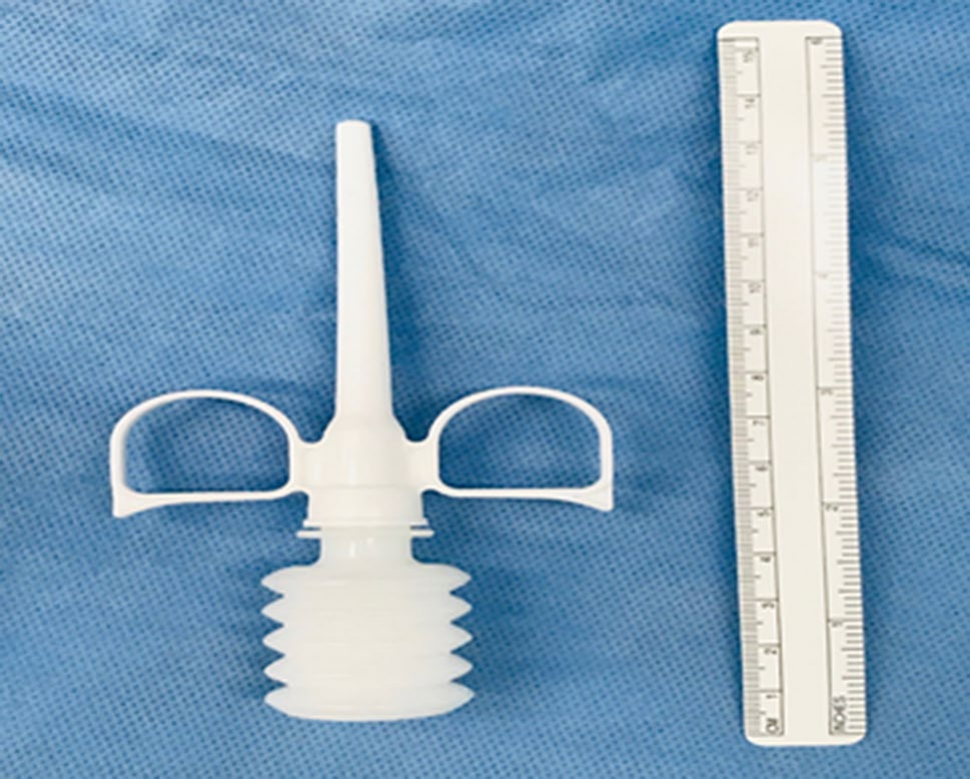
PerClot^®^ Applicator

The application of PerClot^®^, in our practice in our practice, can be explained in three simple steps, under the heading of dry (*D*), apply (*A*) and suction (*S*) clearance. There are a variety of sizes that the surgeon can ask for, 1, 3 and 5 g, with three different applier lengths 100, 200 and 380 mm. In our study, we used 3-g bottles, with a 100-mm applicator (Fig. 1).

**Fig. 2:**
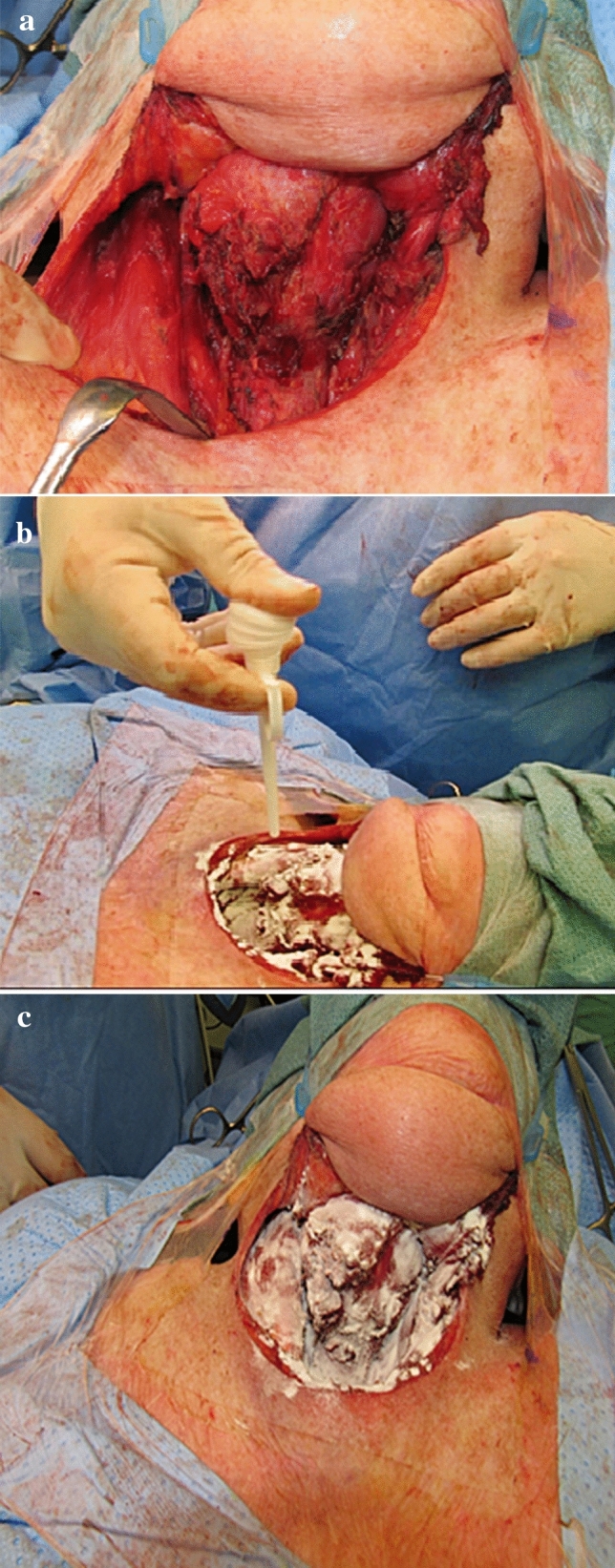
PerClot^®^ in use after open neck surgery: (**a**) Dry (**b**) Application and (**c**) Suction

After connecting the product bottle and the applicator, we dry (*D*) the wound site (Fig. 2a), followed by application of PerClot^®^, while pressing the nozzle all over the wound site (Fig. 2b), and finally after 2 min, we suction (*S*) the excess product leaving a gelled matrix that acts as a mechanical barrier to prevent further bleeding (Fig. 2c).

From our case cohort, we would like to report the adverse events we noticed; these included seroma, haematoma and wound infections related to the use of PerClot^®^. This study showed that 10 out of 57 patients did develop postoperative complications following the use of PerClot^®^ but no allergic adverse effects were noted and it was found safe and easy to use.

Also, in our study, 41 patients out of 57 who underwent head and neck had no surgical drains inserted, thus leading us to report the added haemostatic value of PerClot^®^ which in turn decreased the hospital in-patient stay.

## Conclusion

PerClot^®^ is easy and simple to use. Our study indicates that routine use of a polysaccharide haemostatic agent (PerClot^®^) has a good haemostatic result postoperatively with minimal adverse effects.

Our findings provide evidence for the safe use of PerClot^®^ in common head and neck surgeries, as has been suggested by previous studies in other surgical disciplines. Its use, however, is associated with a small incidence of postoperative wound infection which needs further exploration in future larger blinded studies.
