# Correction to: Use of PerClot^®^ in head and neck surgery: a Scottish centre experience

**DOI:** 10.1007/s00405-020-06328-6

**Published:** 2020-09-05

**Authors:** Kanishka Rao, Anas Gomati, Edwin Yuen Hao Tong, Kim W Ah-See, Muhammad Shakeel

**Affiliations:** Department of Otolaryngology-Head and Neck Surgery, Aberdeen Royal Infirmary, Aberdeen, AB25 2ZN Scotland, UK

## Correction to: European Archives of Oto-Rhino-Laryngology 10.1007/s00405-020-06247-6

In the original publication of the article, the third author name was published incorrectly. The correct name is Edwin Yuen Hao Tong.

The original article was updated.

